# The Philosophy of Marriage, in Its Social, Moral, and Physical Relations: With an Account of the Diseases of the Genito-Urinary Organs Which Impair or Destroy the Reproductive Function, and Induce a Variety of Complaints; with the Physiology of Generation in the Vegetale and Animal Kingdoms

**Published:** 1838-04

**Authors:** 


### ART. VIII.

*The Philosophy of Marriage, in its social, moral, and physical Relations; with an Account of the Diseases of the Genito-urinary Organs which impair or destroy the Reproductive Function, and induce a variety of Complaints; with the Physiology of Generation in the Vegetable and Animal Kingdoms: being Part of a Course of Obstetric Lectures delivered at the North London School of Medicine, Charlotte Street, Bloomsbury Square.* By MICHAEL RYAN, M.D. &c. &c.—*London*, 1837. 8vo. pp. 364.

THIS work is offered, we observe, like several others noticed in this Number of our Review, for the instruction of the public at large; and we once thought of classing it with them: but it must stand by itself; it is in every respect unique. The physiognomy of its title is a true index to its inward character. The Philosophy of Marriage, being pure nomeaning, might have passed without remark; but the remainder of the title-page justifies the strongest condemnation. It is melancholy to see a physician and a man of information descend, from whatever reason, to cater such kind of reading for the ill-regulated minds of all who may choose to indulge in it; offending the eye of delicacy, like a quack-doctor on the wall. And, in the point of ingenuity of attractiveness, even the quack-doctor must yield the palm to Dr. Ryan, with his "account of the Diseases of the Genito-urinary Organs which impair or destroy the Reproductive Function." The work is divided into three parts: the first treats of Marriage, in its moral and social relations, and contains some useful information, intermixed with many topics that look as if they were purely meant to be exciting; the second speaks of the Physical relations of marriage, and may be similarly characterized; the third, which relates to the Morbid relations of marriage, comprehends all the subjects in surgery and midwifery which can offend and disgust, because the information relating to them, whilst it is not useful, pollutes the minds of those who seek it from curiosity, and without any view to its beneficial application. This portion of the work is, we suppose, that alluded to in the title-page as being part of a course of Obstetric Lectures: it contains much that a lecturer would pass lightly over in such a course, even before his medical pupils, and much that, offered to the general public, constitutes an outrage against good manners which nothing can excuse. To attempt to palliate it by quoting Haller as introducing the subject of Generation by saying "there are no secrets in physiology," is as absurd as it would be to make the quotation an apology for walking about the streets of London without clothes, because there are no clothes in physiology, and man is born a naked animal. The author may class those who think as we do on this matter among the "prudish and the ignorant," or "the antiquated and hypocritical," whom he professes a wish to "reconcile" to the consideration of the subjects he dwells upon; and he may affect the air of a virtuous philosopher, while discoursing of the "health of parents and offspring," and the "improvement of morals and population:" but he ought to have been aware of the danger he was running of being confounded with those who, in times past, sought for reputation in the by-paths of literature. Their reward, in their own day, was aversion and contempt—not honour,

> "And unhallowed they sleep in the crossways of fame."

We are greatly deceived if Dr. Ryan will not discover, ere long, that he has committed a grievous mistake, and that neither knowledge nor a facility of composition, nor even an unblemished personal character, can, in the present day, reconcile well-thinking persons to productions which obtrude on modest eyes all that civilized people agree to conceal with decent care.

We have spoken of Dr. Ryan's allusion to Haller in vindication of his own proceedings, although there is nothing in the pure and classic pages of that great physiologist to encourage such writings as the present. He speaks equally vaguely of Aristotle, Spallanzani, and Hunter; and, still evidently a little uneasy about the project he was commencing, says,

> "The period has at length arrived when sexual diseases obtain as much attention as any other class of infirmities, and when the most distinguished medical practitioners devote themselves to their study and treatment. It is scarcely necessary to mention the works of John Hunter, Sir Astley Cooper, Sir Benjamin Brodie, Mr. Guthrie, and many others. *I go a step further*, and describe the moral and physical causes of impotence and sterility, with the physiology and pathology of the sexual organs." (P. 37.)

The mystification displayed in this passage requires no comment; and its motive is obvious: but, thus fortified, the author proceeds to say that "much error exists on the physical laws of reproduction among all classes of society, and especially in relation to the consummation of the marriage contract." He has the modesty to add, that "this requires to be exposed with all the delicacy of which the subject admits, and the precision necessary and privileged in medical works;" (p. 38,) and forthwith a kind of unacknowledged paraphrase follows of a passage in Armstrong's "Economy of Love," giving precise directions as to this delicate particular of consummation! Dr. Ryan might have learned that Armstrong's poem, though full of elegance, was too gross in its subjects for general readers half a century ago, and proved ruinous to the prospects of an experienced and accomplished physician.

The chapter on Premature and Late Marriages contains a few remarks that might be usefully conveyed to popular readers, but mingled with all the idle and fanciful opinions of the ancients,—merely, it would seem, to make up a show. We are told, for instance, that the ancient Germans entertained great respect "for the fair sex;" that a fine of fifteen shillings was levied for baring the arm of a free woman without her consent; that, if her bosom was touched, the fine was forty shillings; and, if a kiss was taken, the punishment was exile: and all this barbarous practice seems to be mentioned with commendation; and we are also favoured with the information that, in the spring of the year 1837, a man prosecuted a woman at the Surrey sessions for biting his nose off; the defence being that he had given the woman a kiss; and that "the judge" said she was justified in biting it off, and would have been justified "in eating it." We can scarcely believe that any judge could disgrace himself by an observation only more disgusting than it is unjust and at variance with all social principles; an observation of such brutal import that it would equally justify murder or any other passionate outrage. But Dr. Ryan's excessive devotedness to the sex makes him admire this ferocious dictum.

When speaking of late marriages, Dr. Ryan informs us that it is "a matter of observation that young women bear no children when united to old men," although observation shews quite the contrary; and in a previous page Dr. Ryan himself seems so struck with the late vigour of men as to be quite ready to affirm, in any disputed case, that there is no physical reason why women also should not propagate at sixty. (P. 34.)

Polygamy and Monogamy are discoursed of with much array of learning. Dr. Ryan quotes Diodorus Siculus, and Xenophon, and Dion Prusæus, to prove that the marriage of brothers and sisters has no good effects: he also adverts to what was said by "Hermione, in his play of Euripides," (a mistake in copying, doubtless;) also to Plutarch, St. Augustin, Vandermonde, Pallas, and Buffon, but without references to passages; and all to prove, what nobody now disputes, that the crossing of breeds is advantageous. We are likewise told at full length the manner of the Grand Seignior's proceeding with a virgin, when allowed one on solemn festivals; and Lady Wortley Montague's account of the eastern mode of dancing is introduced as something piquant on a dull subject: and in this way Dr. Ryan goes on, page after page.

In a chapter on Conjugal and Parental Rights and Obligations, the author dwells at much length on the "conjugal debt," in his peculiar manner; stating the circumstances in which it is to be paid or refused. In what relates to children, he pronounces it barbarous to strike a child under seven years of age, as if striking after that age was quite allowable and proper. He quotes numerous authorities *against* the necessity of fidelity in men,—Samuel Johnson, Napoleon, and Luther;—of course, only to refute them. These instructive remarks precede a very attractive chapter on the Uses of the Sexual Organs; wherein he quotes the expressions of Buffon, "we must describe the history of this age (puberty) with great circumspection, so as not to excite in the imagination other than philosophical ideas:" and he immediately adds, "*we* must treat it in the *pure and unsophisticated state of nature;* that is to say, physiologically." What Dr. Ryan means by physiologically, we shall presently see; but he is evidently too pure, natural, and unsophisticated to dread raising other ideas in the imagination than philosophical ones. He describes the meetings of lovers, their sidelong glances, and the pressure of trembling hands; and at length, he says, "they finally vow to taste legitimate pleasure;" but he very prudently adds, "after swearing eternal fidelity to each other before the altar." He proceeds to give very good advice to young ladies. After some coquetry with the subject of copulation, (p. 135,) he grows shy of it, but very kindly refers the reader, as on many other occasions, to his other works for more precise information, (p. 137.) Not, however, to leave them to burst in ignorance, he tells them why certain organs are called "parts," and why "secret parts;" and adds, with beautiful simplicity, "the unthinking part of mankind consider allusions to these organs as indelicate," (p. 138.) Then he determines to explain coition, in a very matter-of-fact manner; then softens and meanders away into sentimentality again, about the "delights of a conversation full of tenderness;" and then, bolder grown once more, declares his full belief that woman has "a predilection" for a fine figure, a broad chest, a luxuriant growth of hair, and "eyes full of fire," (p. 139.)

In a chapter considerately devoted to unveiling the Causes which influence Fecundity, the author thinks it useful to mention that "many examples" are recorded of married and unmarried women being impregnated when in a profound sleep; and, speaking of antipathy or repugnance as causes opposing impregnation, beautifully remarks "that that which commences with apathy generally finishes with love, when the transport of pleasure ravishes the will." (P. 144.) But in this chapter he particularly lays himself out to be attractive. He enters into the highly useful question of the comparative degree of enjoyment of the sexes in the procreative act; and awards the superiority, for reasons satisfactory to his own mind, although purely fanciful, to the female. With his usual mindfulness of learning, he quotes Plazzonus to prove "unam mulierem pluribus viris sufficere," (p. 152); and he accounts for this supposed fact by an hypothesis equally doubtful. For the instruction of the public in general, he makes some allusion to the subjects of nymphomania and satyriasis, and does not forget to mention cantharides. (P. 154.) To relieve the severe student, he relates the adventure of Proculus, a Roman general, who, having taken an hundred Sarmatian virgins prisoners, writes to a friend "ex his una nocte decem inivi;" and thinking this not enough, he adds the notorious testimony of a lying madman to an achievement of double extent. But then the illustrious Haller says "there are no secrets in physiology."

A chapter, more than half made up of quotations, is devoted to detailing many fanciful opinions concerning the procreation of boys or of girls at will; and another to recounting all the absurd notions that were ever entertained concerning generation; and thus the book swells pleasantly onwards towards a remunerating magnitude. Three more chapters are, however, consigned to the subject of generation, and are interlarded with anecdotes for the instruction of the public mind, very conveniently printed, we conjecture, from the author's lecture-book, as well as the remarks on Pregnancy and Delivery, added with the laudable intention, we suppose, of making every woman her own midwife. But all this supplies materials for 104 more pages.

Yet, this great design being so far accomplished, its candid author, who keeps no secrets, seems to have suffered some solicitude as to whether the book contained all that its predestined readers would require; and the result is, that we have a Third Part, consisting of three chapters, of which the subjects are the pathology of the generative organs; a history of the venereal disease, and an account of urethritis, blennorrhagia, and gonorrhœa. Never before was seen such enthusiasm for the diffusion of useful knowledge among maids, wives, and widows; whose ideas of men, by such a course of instructive reading, must be wonderfully corrected if not purified; and who, thus accomplished, cannot fail to become as interesting to men as men must ever after be in their eyes. The false glosses of poetry, the decent sanctity of prudent concealment, vanish before this rigid declarer of all the secret operations of nature, who, while he thus holds up to view the pleasures and the pains of physical love, adds yet another hundred pages to the Philosophy of Marriage.

When commencing what he calls the pathology of the generative organs, he repeats for the twentieth time that "the human female is unfit to co-operate in the function of procreation until after the twelfth or fourteenth year in this climate," for which he gives, as is his wont, all the reasons, without disguise; adding, in his own manner, "*hence* the cruelty and barbarity of violating female children *of a tender age*" (p. 298); as if the age constituted the crime. He then enters upon the promising field of Impotence, where, of course, he freely expatiates; collecting from every possible authority everything capable of exciting and gratifying the curiosity of the reader. In this, as in all the other portions of his work, there is scarcely an original syllable: the work would be worthless to medical men on this account; for they all know these odd stories as well as Dr. Ryan. The novelty is in all this being laid so bare to the general eye; for what object the compiler best knows. One instance of his modesty, however, we will not conceal. After revelling in stories of impotence through thirteen pages, he says, "it would be contrary to the national taste and propriety to give a detail of the numerous diseases caused by the *abuse* of the reproductive function" (p. 311); and then goes on to enumerate them, and fills a whole page with evils greater than Thersites ever collected into an evil wish, including "incarceration of the penis in foreign bodies:" and he says he might give *several* illustrations of *each* of the hundred affections he mentions, so that his reserve and modesty on this occasion are too singular to be passed over. After this self-denial, Dr. Ryan gives the reader seven and twenty more pages full of further capital stories about impotence!

The most edifying thing of all is, that, after finishing this ill-arranged, offensive, and very indifferently written work, the philosophic author concludes it like a sermon. "I now submit these pages," he says, "the result of many years' study, reflection, and observation, to an enlightened age; impressed with the conviction that they are calculated to correct much error, and with sanguine hopes that they may benefit the present and succeeding generations." This is indeed admirable, but only for its unfitness as the conclusion of such a book, in the production of which Dr. Ryan has lamentably forgotten what is due to the public, to the profession, and to himself.

